# Peat: A Natural Source for Dermatocosmetics and Dermatotherapeutics

**DOI:** 10.4103/0974-2077.53094

**Published:** 2009

**Authors:** Uwe Wollina

**Affiliations:** *Department of Dermatology and Allergology, Hospital Dresden-Friedrichstadt, Academic Teaching Hospital of the Technical University of Dresden, Dresden, Germany*

**Keywords:** Fulvic acids, humic substances, peat

## Abstract

In recent years the interest for natural substances in dermatotherapy and cosmetics has increased. Peat is a complex natural source of humic substances that are of potential interest in both dermatology and cosmetology. Humic substances in peat have been partially characterized and pharmacologic and biologic activities have been documented. Possible clinical applications are outlined.

## INTRODUCTION

Natural substances have historically been a rich source of lead molecules in drug development. Recently, effective high-impact technologies have become available to screen natural products.[1] There is a growing serious interest in characterization of natural substances in medicine, in particular dermatology, and dermocosmetics.[2,3]

Peat, a source of natural substances, has long been used in physiotherapy, rheumatology and sports medicine. Peat is a rich natural source of humic substances that are of potential interest in dermatology and cosmetology.

**Figure 1:**
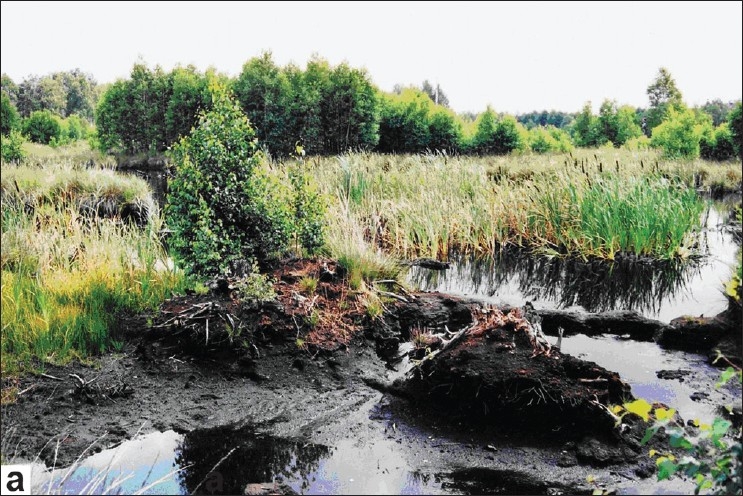
Natural peat from the Altteicher Moor (Germany)

Humic substances are natural products that develop during decomposition of organic matter in humus. Humic substances are the most stable fraction of organic substances in soils. The dark color comes from quinine structures [Figure 1]. A substantial part of humic acids is in carboxylic acid functional groups that bind multivalent positively charged ions.[4] Although the composition of peat is very complex and not completely understood[5,6] the principal substances may be divided into the following categories:[7]

- humic acids
- humin
- fulvic acids

Other major components include pectins, cellulose and lignins, wax, resins and inorganic material. Dark peat contains about 10 times more humic substances than the white garden peat. The composition and the amount of the particular ingredients may vary with the geographical area, where peat was harvested. Drying peat can cause a breakdown in the natural cellular structure that negatively interferes with its water solubility.

Humic substances are highly chemically active yet recalcitrant with respect to biodegradation. They have several biologic activities such as antibacterial, antifungal, immunomodulatory and photoprotective actions that are of potential interest in dermatology and cosmetology.

## STUDIES ON THE PHARMACOLOGIC AND BIOLOGIC ACTIVITY OF PEAT

In the past, crude extracts from peat of various sources have been screened for antibacterial activity.[8] Humic acid and fulvic acid exert antimicrobial activities *in vitro*.[9] Antibacterial activity has been shown against a variety of pathologic germs including *Staphylococcus aureus*, *Escherichia coli*, *Pseudomonas aeruginosa*, *Klebsiella pneumoniae* and against yeasts like *Candida albicans*.[9,10] Humic acids and poly(OH)carboxylates are selective inhibitors of *Herpes simplex virus* and Cytomegalovirus replication.[11,12] Oxihumate – a water-soluble compound of peat – inhibited HIV-1 infection of MT-2 cells with an IC(50) of 12.5 μg/ml. Treatment of free and cell-attached HIV with oxihumate irreversibly reduced infectivity, while the susceptibility of target cells to the virus was not impaired by treatment prior to infection. The infectivity of the HIV particles was inhibited by interference with CD4 binding and the V3 loop-mediated step of virus entry. No viral resistance to oxihumate developed over a 12-week period *in vitro*.[13] Furthermore, it was shown *in vivo* that oral administration of humus extract in freshwater fish prevents infection with the fish pathogen *Aeromonas salmonicida*.[14] The remnants of a biodiversity of plants are responsible for the broad-range antimicrobial activity found in peat.[15,16]

Humic substances show a wide range of other activities[17] [Table 1]. A low molecular weight preparation of peat (Tolpa Torf Preparation-TTP) induces tumor necrosis factor-alpha (TNF-α) and interferon (IFN)-α and -γ in human peripheral mononuclear blood cells *in vitro*.[18] Immunoprotective activity has been suggested from a study of oral treatment of athletes with TTP.[19] The effects of oxihumate on the proliferative response of lymphocytes have been studied *in vitro* and *ex vivo*. Oxihumate increased the proliferative response of phytohemagglutinin-stimulated human lymphocytes, from a concentration of 20 μg/ml and upwards. This response was even more striking in the case of lymphocytes from HIV-infected patients and similar effects were observed *ex vivo* following administration of a non-toxic dosage of 4 g oxihumate per day to HIV-positive individuals for two weeks. Studies revealed that stimulation of the proliferative response of lymphocytes by oxihumate is associated with an increased production of IL-2, as well as expression of the IL-2 receptor in the setting of decreased production of IL-10. Oxihumate therefore holds promise for the treatment of immunocompromised patients.[20]

**Table 1:** Documented activities of humic substances[17]

Adsorptive
Astringent
Agonistic effect on α2-adreno and dopamine (D2) receptors
Antiallergic
Antibacterial
Anticoagulatory
Anti-inflammatory
Antiviral
Chelators for heavy metals
Estrogenic
Hemostyptic
Hyperemic
UVB-protective

Of particular interest is the UVB protection by peat substances. Humic acids were investigated *in vitro* using U937 culture cells. The cells were exposed to UVB and UV-induced cytotoxicity was assessed with the XTT reduction assay (EZ4U, Biozol). Ammonium humate and humic acids showed a protective activity by UVB absorption that is in the range of *p-aminobenzoic acid.*[21]

High-pressure liquid chromatography (HPLC) analysis revealed that aqueous peat extracts contain up to 18 fractions of water-soluble compounds of fulvic and humic acids. These compounds have been found to have a stimulatory response on the contractile activity of smooth muscles. *In vitro* diffusion studies showed that the permeability of these substances across human full-thickness skin (thickness: 200 μm^−1^) is highly selective and the resulting stimulatory activity is dependent on the permeated fraction. Especially, the HPLC fractions 7-11 and 14 are able to permeate human skin. Fractions 7-11 show a moderate stimulatory effect on smooth muscles for more than 90 min whereas fraction 14 shows the strongest stimulatory effect which was, however, suppressed after 87 min. These results show that the cutaneous therapy with peat treatment results in transcutaneous permeation of biologically active fulvic and humic acid derivatives explaining the additional pharmacological effect of peat treatment in clinical practice.[22] In experimental rodent studies, therapeutic bathing in peat and humic acids extracted from peat resulted in significant reduction of intraabdominal gynecological adhesions.[23]

Rats treated with natural peat or isolated humic acids showed a decrease of total cholesterol, total lipids, an increase of the high-density lipoprotein fraction, and decrease in glucose levels. Furthermore, there was an increase of immunoglobulins, erythrocytes, hemoglobin, and hematocrit after 24 days of treatment.[24]

## POTENTIAL INDICATIONS OF HUMIC SUBSTANCES IN DERMATOLOGY AND COSMETIC DERMATOLOGY

The use of peat has a long tradition in spa medicine and rheumatology. Whereas complete peat is mostly used under these circumstances, peat extracts and isolated humic substances are of greater interest in dermatology and cosmetology.

Because of mild astringent and anti-inflammatory activity, humic substances are useful adjuncts in the topical therapy of inflammatory skin diseases like atopic dermatitis, cheiropodopompholyx, psoriasis and mild focal hyperhidrosis.

**Figure 2:**
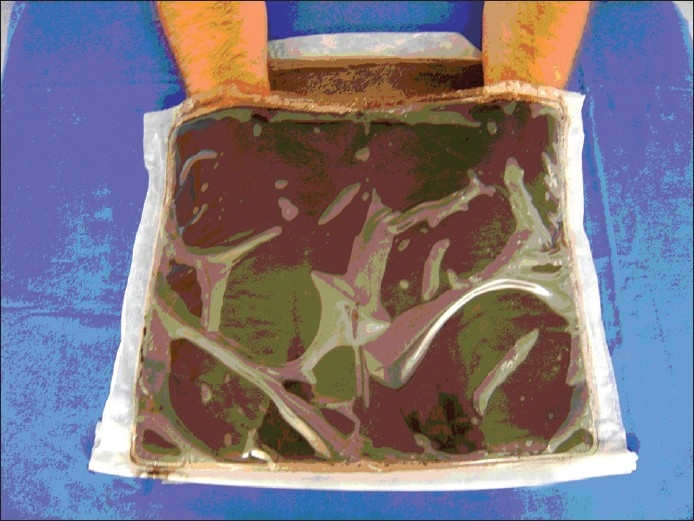
Peat application in chronic hand dermatitis

Preparations of humic acids and other peat compounds can be specifically designed for use on the body, scalp or palmoplantar skin. The compounds allow incorporation in gels (e.g. shower gels), creams and ointments, and may be used for bath therapy as well. An open trial with daily peat application showed benefit in patients suffering from chronic hand dermatitis and psoriasis palmaris.[25] Peat from the Altteicher Moor (Germany) was used as an adjunct to standard therapy [Figure 2]. Patients reported a more rapid relief from itch, inflammation was decreased and pustule formation was stopped earlier than usual. Astringent activity of humic substances may be responsible for such an effect.[25]

The efficacy of humic substances in impetigo has not been studied. The antibacterial spectrum of *in vitro* activity, however, would suggest a possible role.

Another potential indication is candidal intertrigo. Both anti-yeast activity and astringent activity could favor a clinical response in this often hard-to-treat condition.

In skincare products, they are tolerated even by sensitive skin. Their UVB-protective activity might need higher concentrations than usually available in the natural peat sources.

Peat applied topically may exert systemic effects by absorption of humic substances. Water-soluble compounds of fulvic and ulmic acids have been found to have a stimulatory response on the contractile activity of smooth muscle by dopaminergic D2 and α2-adrenergic stimulation.[22] D2-receptors are involved in opioid-induced pruritus.[26] Peat baths might be beneficial in these patients, but no studies have been performed yet.

Humic substances may be of potential interest in rosacea. Here, flushing and inflammation are mediated by neurogenic mediators.[27] Serotonin uptake inhibitors and α-adrenergic agonists have been shown to suppress erythema and flushing.[28,29] Humic substances have a multimodal function in this disease, since UVB protection would inhibit a major trigger and α2-adrenergic receptor blockade might provoke a better α2-response.

**Figure 3:**
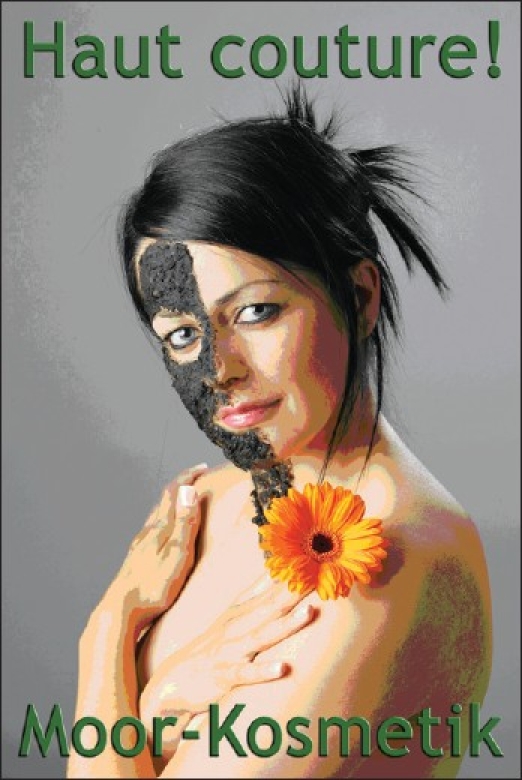
Dermocosmetics based on natural peat

Since humic acids have shown antiviral activity *in vitro*,[11] their use in facial masks for prevention of viral reactivation after chemical or laser peeling would be of interest [Figure 3]. Topical application in uncomplicated cases of cutaneous herpes simplex warrants clinical trials.

Among anti-ageing factors, plant sterols have been of particular interest for female cosmetics. Sterol extracts from peat have been marketed in facial creams. Exfoliative activity of peat is used in bath foams and shower gels. Anti-inflammatory activity is used in toothpastes and gels. The “detoxifying” activity of topical peat is a myth not substantiated by human studies.

No measurable side-effects have been observed so far.[30,31]

## CONCLUSION

It is obvious that peat is a source of several chemicals which can find uses in dermatology and cosmetic dermatology. Further studies are needed to elucidate their actions and efficacy so that proper formulations can be designed for clinical use.
